# Mycobacterium tuberculosis, Streptococcus pneumoniae, and Staphylococcus aureus Co-infection in a Two-Year-Old Immunocompetent Patient: A Case Report

**DOI:** 10.7759/cureus.84063

**Published:** 2025-05-13

**Authors:** Giorgie Petković, Ivana Maloča Vuljanko, Zrinka Kačić Miličić, Ana Tripalo Batoš, Ivan Pavić

**Affiliations:** 1 Department of Pulmology, Children's Hospital Srebrnjak, Zagreb, HRV; 2 Department of Radiology, Children's Hospital Zagreb, Zagreb, HRV; 3 Department of Pediatrics, Children's Hospital Zagreb, Zagreb, HRV

**Keywords:** bacterial, children, co-infection, necrotic, pleuropneumonia, tuberculosis

## Abstract

Co-infections in pulmonary tuberculosis are rare among immunocompetent children in settings with low tuberculosis prevalence. We present a case of a two-year-old immunocompetent child with necrotic pleuropneumonia caused by *Mycobacterium tuberculosis*, *Streptococcus pneumoniae*, and *Staphylococcus aureus* co-infection in a low tuberculosis prevalence setting.

A 24-month-old boy presented with a five-day history of cough, followed by three days of high fever and dyspnea. Initial laboratory inflammatory markers were elevated. Chest ultrasound, radiography, and CT scan revealed necrotic changes in the right upper lobe with bullae, pleural effusion, and subcutaneous emphysema. Initial therapeutic procedures included the evacuation of 140 mL of hemorrhagic content from the pleural space. Microbiological analysis revealed *Streptococcus pneumoniae* type 3 from pleural effusion and *Staphylococcus aureus* from blood culture. Antimicrobial therapy included ceftriaxone and clindamycin for six weeks. Following flexible bronchoscopy, microbiological culture from aspirated material detected *Mycobacterium tuberculosis*. Anamnesis did not clarify any prior contact with a tuberculosis-infected individual.

This case represents an example of *Streptococcus* and *Staphylococcus* superinfection on evolving pulmonary tuberculosis. To our knowledge, no literature is currently available indicating the co-existence of tuberculosis, streptococcal, and staphylococcal pulmonary infection in an immunocompetent patient in a population with low tuberculosis prevalence.

## Introduction

Necrotizing pneumonia (NP) is an uncommon, severe complication of pneumonia, and there has been a gradual increase in its incidence. This rise is partially explained by greater physician awareness, the use of contrast-enhanced CT scans, temporal changes in circulating respiratory pathogens, and antibiotic overuse. The most common pathogens detected in children with NP are *Streptococcus pneumoniae* and *Staphylococcus aureus*. Cases caused by *Streptococcus pneumoniae* are declining due to vaccination, while cases of *Staphylococcus aureus* are rising in frequency [1]. Clinically, NP is characterized by progressive pneumonic illness in a previously healthy child despite appropriate antibiotic therapy and typically follows a protracted clinical course [2,3].

Tuberculosis (TB) is a communicable disease caused by *Mycobacterium tuberculosis* that remains a major public health concern worldwide [4]. TB incidence in developed world has been decreasing in recent years, but targets for eliminating TB have still not been achieved in many developed countries.

Literature review revealed probably the first article which mentioned secondary infection in pulmonary TB, published in 1911 [5]. Since then, only few articles of TB and bacterial co-infection in children have been published. Simultaneous co-infection is considered rare especially in an immunocompetent individual [6]. However, in high burden countries where TB is known to be endemic, TB and other bacterial co-infection are more common, especially in HIV infected or otherwise immunodeficient children [7-9]. One of the reasons for rarity of this diagnosis could be the similarities in the symptoms and signs of TB and bacterial NP, that make it difficult to distinguish those two entities [7].

## Case presentation

The patient is the fifth child in the family, born after a normal pregnancy and with no history of serious illnesses. The patient received the Bacillus Calmette-Guérin (BCG) vaccine in infancy. Vaccination against pneumococcal disease was performed with a 10-valent vaccine in two doses according to the vaccination schedule, while the third dose was administered after the described case. The maternal grandfather is a kidney patient, but there are no other serious chronic diseases in the family. They live in a house and have a horse in the yard. Three weeks before the onset of the boy's illness, his older sister had a respiratory illness.

At the time of presentation, the boy was two years old, with development and nutrition appropriate for his age. The first clinical sign, cough, appeared five days before hospitalization. Three days before hospitalization, the boy developed fever, dyspnea, and loss of appetite.

**Figure 1:**
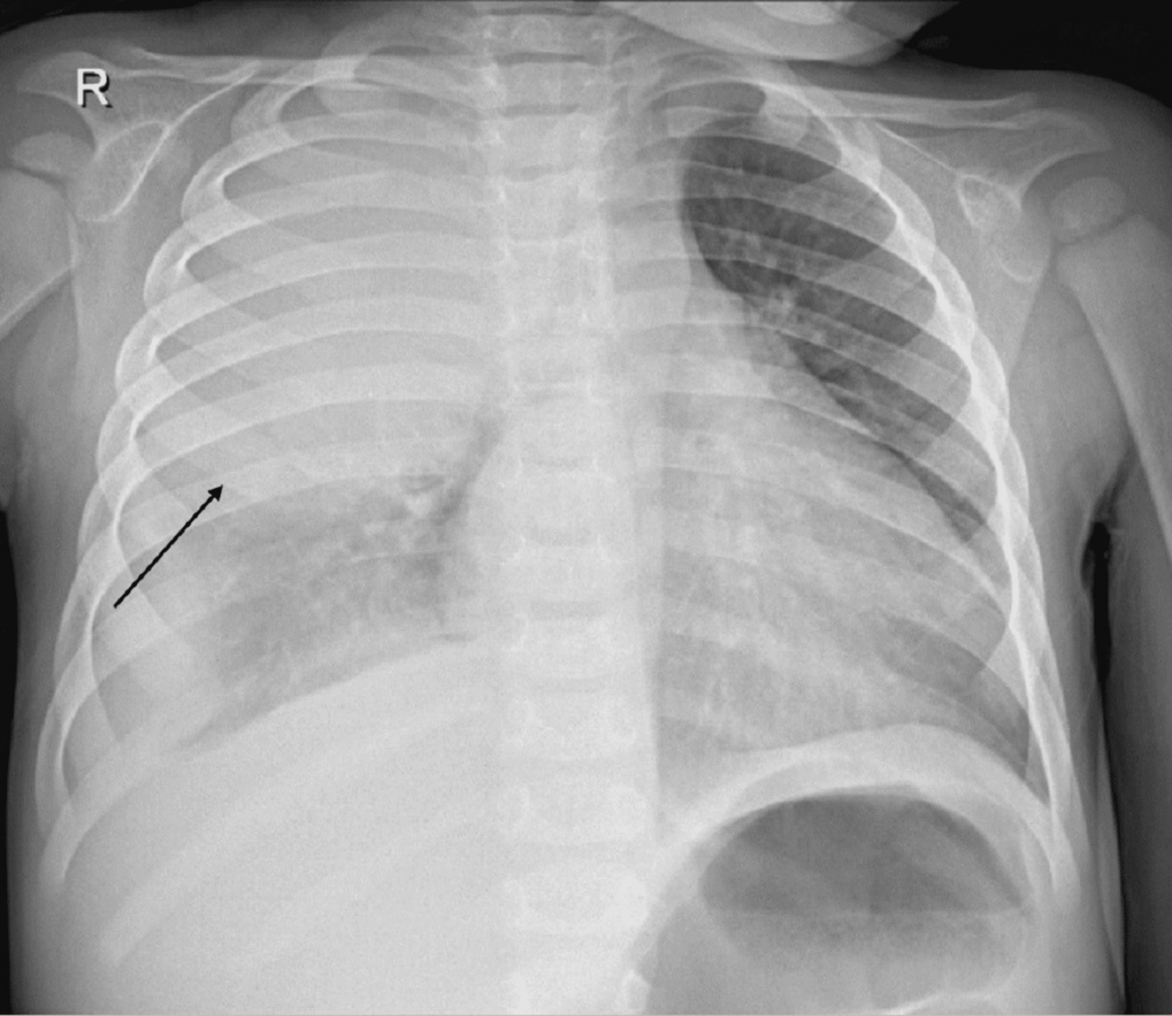
Chest X-ray at hospital admission (AP erect view). Homogeneous opacity of the upper two-thirds of the right hemithorax with left mediastinal shift. Inhomogeneous opacity in the basal part of the right lung indicates pleural effusion. AP: Anteroposterior.

Initial workup included a chest radiograph (X-ray) at hospital admission, which showed homogeneous opacity of the upper two-thirds of the right hemithorax with left mediastinal shift and inhomogeneous opacity in the basal part of the right lung, indicating pleural effusion (Figure 1).

Inflammatory markers were elevated (blood leukocytes 28.69×10⁹/L; C-reactive protein 145 mg/L; procalcitonin 7.8 μg/L). A throat swab was negative for group A beta-hemolytic Streptococcus (BHS-A), and a nasopharyngeal aspirate tested by polymerase chain reaction (PCR) for SARS-CoV-2, respiratory syncytial virus (RSV), adenovirus, and influenza virus did not demonstrate the presence of these pathogens. *Streptococcus pneumoniae* type 3 was isolated from the pleural effusion by culture (antibiogram: ampicillin S, ceftriaxone S, moxifloxacin S, erythromycin S, azithromycin S, sulfamethoxazole-trimethoprim S, tetracycline S, amoxicillin S, penicillin S). *Mycobacterium tuberculosis* was not demonstrated in the effusion, and adenosine deaminase was not performed. Upon admission to the hospital, a blood culture was performed from which *Staphylococcus aureus* was isolated (antibiogram: ampicillin R, cloxacillin S, amoxicillin-clavulanic acid S, gentamicin S, ciprofloxacin I, azithromycin R, clindamycin S, sulfamethoxazole-trimethoprim S, vancomycin S, tetracycline S, penicillin R). Isolated pathogens were sampled after the first dose of ceftriaxone.

Treatment began with pleural drainage, which removed 140 mL of hemorrhagic effusion, and alteplase 0.1 mg/kg was administered for two days during the course of drainage. Antimicrobial treatment was initiated on the first day with ceftriaxone 650 mg twice daily, and clindamycin 140 mg three times daily was added to the therapy on the fourth day upon receipt of the blood culture results, for the purpose of treating the identified pathogens.

**Figure 2:**
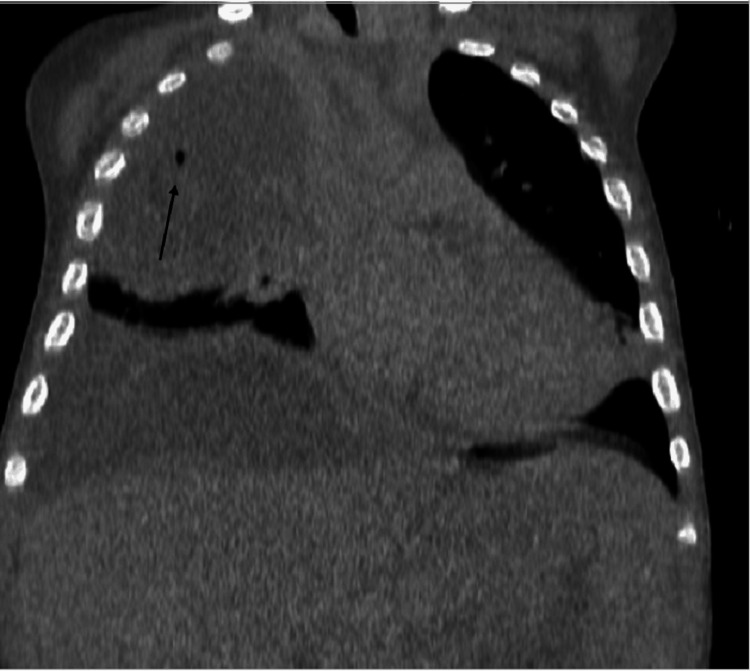
Post-contrast MSCT coronal reconstruction. Consolidation of the lung parenchyma in the upper right lobe with gas bubbles within the consolidation, suggesting necrosis. Mild left-sided shift of the mediastinum. Right-sided hemorrhagic subpleural effusion. MSCT: Multi-slice computed tomography.

Pulmonary status was monitored by ultrasound examinations and X-ray. Due to the severity of pulmonary changes, including necrosis and effusion, CT diagnostics was performed to evaluate the extent and progression of the pathological process. On the seventh day of hospitalization, post-contrast MSCT coronal reconstruction showed consolidation of the upper right lung lobe with gas bubbles inside the consolidation suggesting necrosis, a mild left-sided mediastinal shift, and a right-sided hemorrhagic subpleural effusion (Figure 2).

On the seventh day after admission, non-bronchoscopic bronchoalveolar lavage was performed, which returned sterile. The clinical picture was further complicated by the formation of bullae in the destroyed parenchyma, which led to the development of subcutaneous emphysema on the right side of the chest.

The boy became afebrile on the eighth day of hospitalization (after eight days of ceftriaxone and four days of clindamycin therapy), with a decrease in inflammatory markers. On the tenth day of treatment, blood leukocytes were 15.4×10⁹/L, C-reactive protein 19.1 mg/L, and procalcitonin 0.21 μg/L; after four weeks of therapy, blood leukocytes were 15.8×10⁹/L, C-reactive protein 1.2 mg/L, and procalcitonin 0.06 μg/L (Table 1).

**Table 1:** Inflammatory markers. The table shows the decrease in inflammatory markers during the course of therapy. CRP: C-reactive protein.

Marker	At Admission	After 10 Days	After 4 Weeks	Normal Range	Unit
Leukocytes	28.69	15.4	15.8	6.0-16.0	×10⁹/L
CRP	145	19.1	1.2	0.1-2.8	mg/L
Procalcitonin	7.8	0.21	0.06	<0.10	μg/L

There was a decrease in pleural effusion, and the pleural drain was removed after ten days of treatment, with continued radiologic monitoring.

After three weeks, flexible bronchoscopy was performed to repeat bronchoalveolar lavage for microbiological and cytological evaluation and to inspect the bronchial tree for any anatomical anomalies. The lavage revealed 95% neutrophils. The sample was microscopically negative for acid-fast bacilli, but *Mycobacterium tuberculosis* was isolated from the culture on the second day (resistance test: negative for streptomycin, isoniazid, rifampicin, ethambutol, and pyrazinamide), and treatment for pulmonary tuberculosis was initiated. The initial intensive phase of tuberculosis therapy included isoniazid 140 mg, rifampicin 200 mg, pyrazinamide 470 mg, and ethambutol 270 mg, all administered orally. An interferon gamma release assay (IGRA) was performed twice using the QuantiFERON-TB Gold Plus test method, once during hospitalization and again two months after discharge. Both tests were negative (interferon-gamma CD4 and CD4/CD8 responses in both samples were <0.05 kIU/L; positive control was >0.5 kIU/L) (Table 2) [10].

**Table 2:** Quantiferon-TB Gold Plus test. The table shows the results of the Quantiferon test along with the values of the positive controls.

Marker	1st Examination	2nd Examination	Normal Range	Unit
Interferon-gamma CD4	< 0.05	< 0.05	< 0.35	kIU/L
Interferon-gamma CD4/CD8	< 0.05	< 0.05	< 0.35	kIU/L
Positive control	> 10	> 10	≥ 0.5	kIU/L

The test was performed as a routine investigation and is not used to confirm active pulmonary tuberculosis. Due to these findings, an epidemiological investigation of close contacts was carried out, which did not identify the source of infection (i.e., no “zero patient” was identified in the environment).

**Figure 3:**
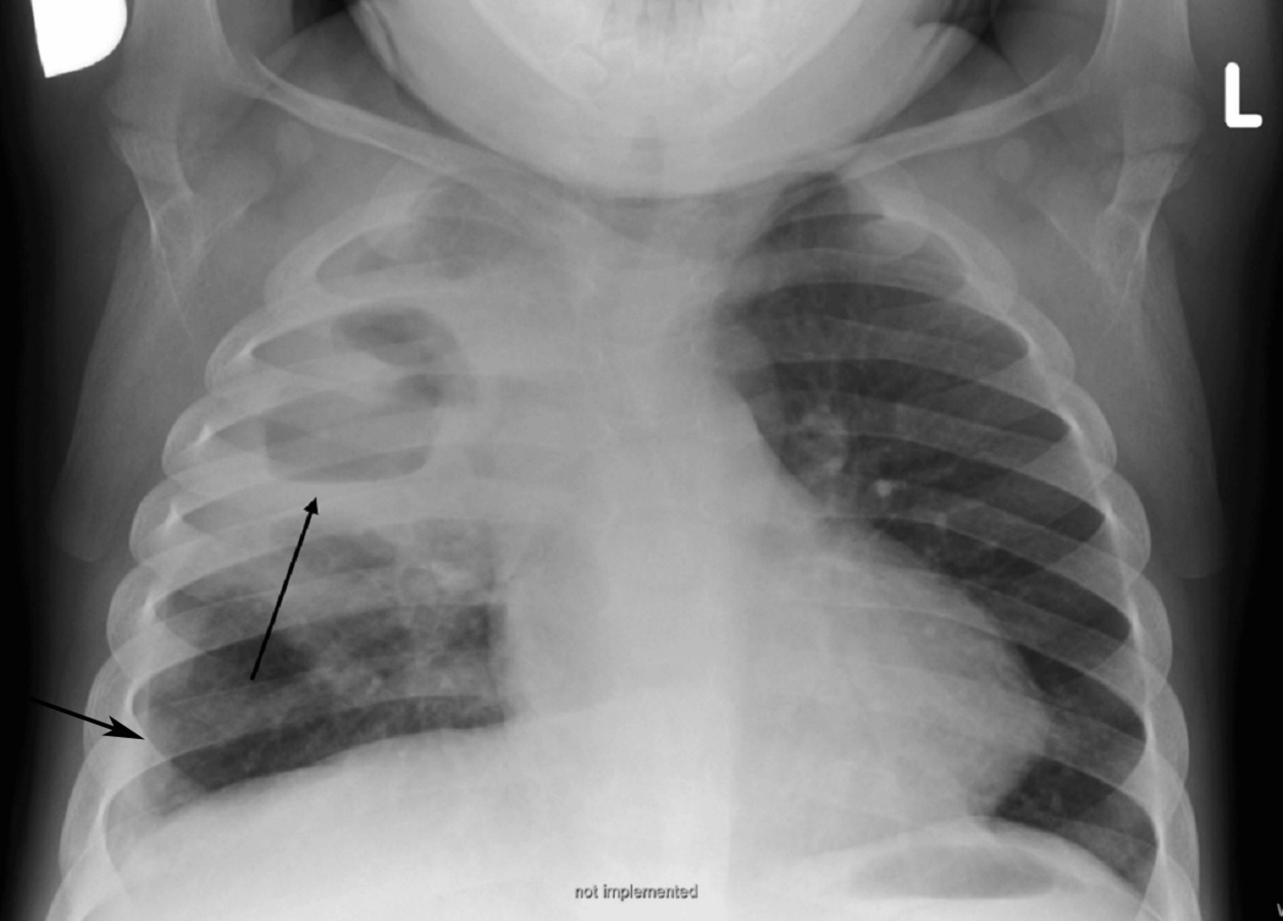
Chest X-ray at the time of discharge (AP erect view). On the right, an almost homogeneously shadowed upper half of the lung is seen, with a central transparency zone corresponding to an abscess cavity (without a true air-fluid level) or bulla. Adjacent to the right lateral thoracic wall, thickening of the pleural space is visible, suggestive of pleural thickening or discrete effusion. AP: Antero-posterior.

The patient was discharged after six weeks of antimicrobial treatment with ceftriaxone and clindamycin. A chest X-ray at the time of discharge showed an almost homogeneous shadow in the upper half of the right lung, with a central zone of transparency possibly corresponding to an abscess cavity (without a definitive air-fluid level) or bulla. Adjacent to the right lateral thoracic wall, thickening of the pleural space was noted, possibly representing pleural thickening or discrete effusion (Figure 3).

**Figure 4:**
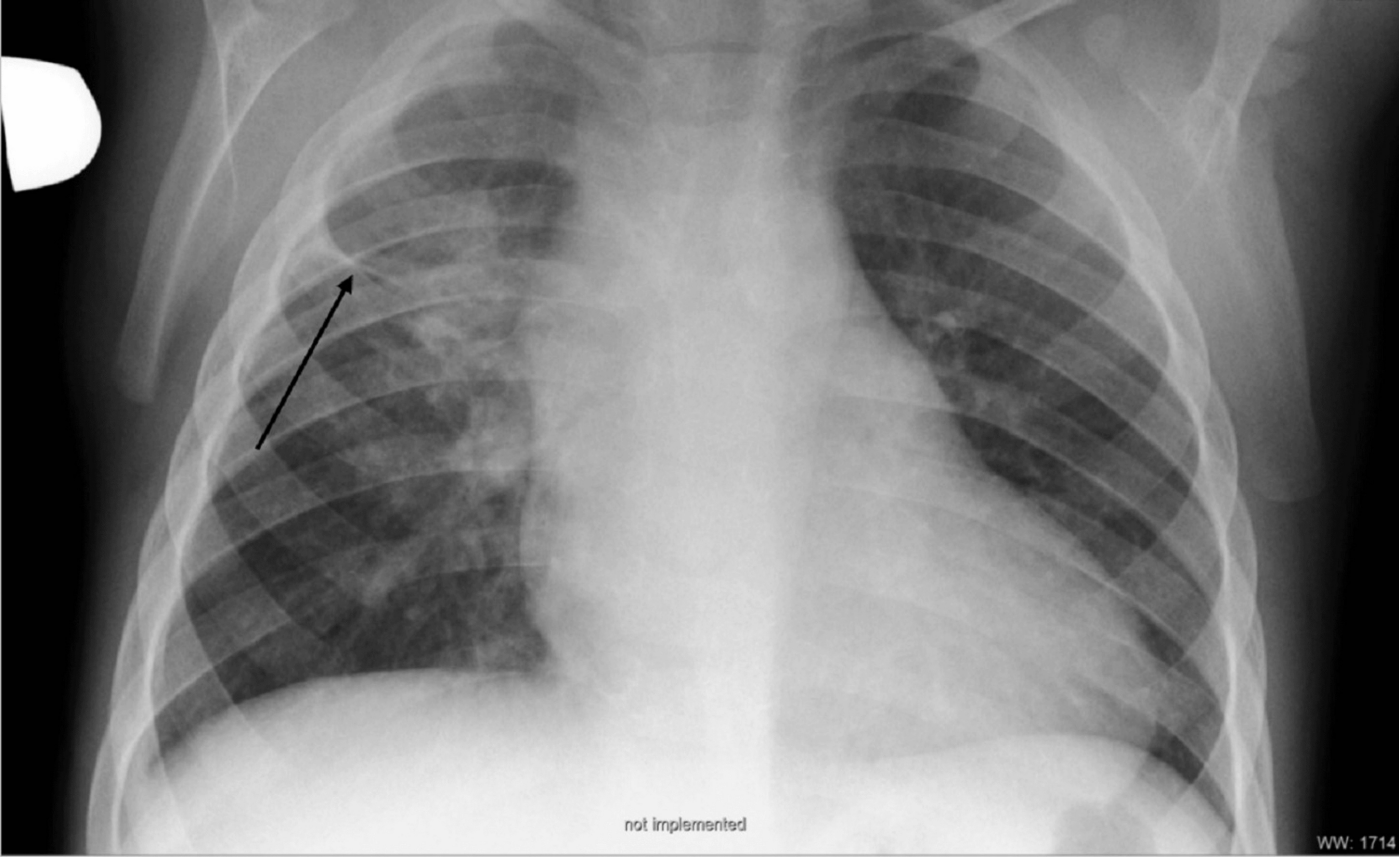
Chest X-ray four months after discharge (AP erect view). On the right, in the area of prior inflammation, a fibrous zone is visible along with a more cranially positioned horizontal interlobium, consistent with scarring. AP: Anteroposterior.

The boy underwent intensive antituberculosis therapy for two months. Three control gastrolavages were performed, all of which were microscopically negative for acid-fast bacilli and culture-negative for *Mycobacterium tuberculosis*. Upon receipt of these findings, consolidation therapy for pulmonary tuberculosis with isoniazid 140 mg and rifampicin 200 mg orally was continued for another four months. The boy tolerated the treatment well and experienced no side effects. Upon completion of the full treatment course, complete clinical recovery was recorded, along with gradual normalization of heart and lung radiographs. A chest X-ray performed four months after discharge showed, on the right side in the area of prior inflammation, a fibrous zone along with a more cranially positioned horizontal interlobium consistent with scarring (Figure 4).

The immunological examination was unremarkable. Immunoelectrophoresis performed on two occasions showed the following values: IgA 1.57 and 0.9 g/L, IgM 1.07 and 0.9 g/L, and IgG 12.25 and 8.5 g/L. IgG subclass measurements also remained within reference ranges on both occasions: IgG1 5.83 and 7.21 g/L, IgG2 1.28 and 0.89 g/L, IgG3 0.512 and 0.937 g/L, and IgG4 0.104 and 0.086 g/L (Table 3). Immunophenotyping of peripheral blood lymphocytes did not indicate immunodeficiency.

**Table 3:** Immunoelectrophoresis and IgG subclasses. The table shows the results of immunoelectrophoresis and IgG subclass examinations.

Marker	1st Examination	Normal Range	2nd Examination	Normal Range	Unit
IgA	1.57	0.5-1.3	0.9	0.3-1.7	g/L
IgM	1.07	0.5-1.9	0.9	0.4-1.6	g/L
IgG	12.25	5.0-13.6	8.5	3.0-10.7	g/L
IgG1	5.83	2.70-9.40	7.21	2.80-13.70	g/L
IgG2	1.28	0.44-1.90	0.89	0.44-3.00	g/L
IgG3	0.512	0.090-0.630	0.937	0.130-0.840	g/L
IgG4	0.104	0.023-0.590	0.086	0.005-1.140	g/L

The anti-HBs titre was negative. The mitogenic positive control of lymphocyte proliferation with phytohemagglutinin, as part of the QuantiFERON test, was normal on both occasions, with values exceeding 10 kIU/L (normal range ≥0.5 kIU/L) (Table 2) [10]. Chloride concentration in sweat was below 10 mmol/L (normal range: <30 mmol/L). The boy is currently healthy and has had no serious respiratory or infectious illnesses. Due to the specificity of the clinical presentation, he is being monitored by an immunologist.

## Discussion

This case report presents a rare clinical scenario involving a two-year-old immunocompetent child with necrotic pleuropneumonia caused by *Mycobacterium tuberculosis*, *Streptococcus pneumoniae*, and *Staphylococcus aureus* in a low tuberculosis incidence setting (3.9 per 100,000 in 2021) [11].

A review of the literature indicates that pulmonary tuberculosis increases the risk of secondary bacterial pneumonia in populations with high TB burden and/or HIV immunodeficiency [7, 12, 13, 14], or in older patients with comorbidities [15]. It is worth noting that in Asian countries, as much as 7% of community-acquired pneumonia cases are re-diagnosed as pulmonary tuberculosis [16]. In contrast, our patient lived in a low TB burden setting and had no immunodeficiency. Co-infections with *Mycobacterium tuberculosis* and other bacterial pathogens in immunocompetent individuals, especially in areas with low TB prevalence, are quite rare. The presented case provides a unique perspective on the challenges of diagnosing and managing co-infections involving *Mycobacterium tuberculosis*, *Streptococcus pneumoniae*, and *Staphylococcus aureus* in an immunocompetent child, particularly in a region with low TB prevalence.

Secondary infections in pulmonary tuberculosis are known to increase morbidity and mortality in TB patients, often presenting with significant leukocytosis and more rapid, severe clinical progression [5]. A study from South Africa reported that 43% of children with pulmonary tuberculosis had symptoms of acute pneumonia [17]. A similar rapid and severe clinical manifestation was observed in our patient. The clinical presentation of the two-year-old boy, including cough, fever, dyspnea, and loss of appetite, demonstrates the severity of the disease. Imaging findings of homogeneous consolidation, effusion, and subsequent liquefaction of lung parenchyma with gas bubbles and aero-liquid levels highlight the necrotizing nature of the pneumonia. The formation of subcutaneous emphysema further added to the complexity of the case. The rarity of such cases, especially in areas with low TB incidence, underscores the diagnostic and therapeutic challenges posed by these infections. The possible acute manifestations of pulmonary tuberculosis include acute progressive TB, subclinical TB with bacterial or viral superinfection, or an initial bacterial/viral pneumonia that increases susceptibility to *Mycobacterium tuberculosis* [18]. The diagnostic dilemma arises from the overlapping clinical symptoms of TB and bacterial pneumonia, making it crucial to conduct a thorough diagnostic workup, including microbiological analysis and imaging studies.

Furthermore, 49% of hospitalized tuberculosis patients have been shown to respond to first-line antibiotic treatment for community-acquired pneumonia [7], as was the case in our patient. His initial treatment included a combination of antibiotics and antitubercular therapy, leading to a positive outcome after six weeks of treatment. This underlines the importance of rapid and appropriate management in complex TB cases such as this one.

The most common causes of bacterial co-infection in tuberculosis patients are *Streptococcus pneumoniae* and *Haemophilus influenzae* [8]. One study reported that 10% of culture-positive tuberculosis patients had a positive hemoculture [7]. Our patient had a dual bacterial co-infection: *Streptococcus pneumoniae* was isolated from pleural effusion, and Staphylococcus aureus from blood culture. This co-infection is notable, as both bacteria are known to cause severe pneumonia with abscess formation and lung necrosis [3, 19]. The finding of bacteremia is also significant, considering how difficult it can be to diagnose bacterial co-infections in pneumonia. Moreover, to our knowledge, combined superinfection with Staphylococcus aureus and *Streptococcus pneumoniae* in a tuberculosis patient has not been previously reported. Distinguishing between tuberculosis and bacterial pneumonia can be challenging due to overlapping clinical symptoms. This case highlights the importance of a comprehensive diagnostic workup, including microbiological analysis and imaging studies, to accurately identify the causative pathogens.

In 2020, Saldaña NG et al. [6] reported a co-infection with* Streptococcus anginosus *and *Mycobacterium tuberculosis* in an 11-year-old boy. Similar to our case, the patient was immunocompetent, lived in an area with a tuberculosis incidence of 9.1 per 100,000 population per year, had no known household or close contacts, and presented with acute and severe symptoms, including a right lower lobe lung abscess and empyema. However, that patient had persistent symptoms despite antibiotic treatment and drainage, whereas in our case, there was clear clinical improvement after initial treatment. Furthermore, the most striking difference is that our patient had a polymicrobial superinfection involving two distinct bacterial pathogens. Comparisons with similar cases in the literature, such as the one reported by Saldaña NG et al., provide valuable insights into the diverse clinical presentations of such co-infections. McNally LM et al. [20] reported that polymicrobial infections are relatively common in infants with pneumonia. In their study, 15% of infants with acute pneumonia who did not respond to initial antibiotic treatment were found to have *Mycobacterium tuberculosis*. Among these children, 85% had acute symptoms lasting less than two weeks. The study also noted that maternal tuberculosis was associated with severe pneumonia in infants, particularly when compounded by factors such as young age, multiple pathogens, and maternal HIV status.

## Conclusions

This case report adds valuable information to the existing medical literature, emphasizing the need for vigilance and a comprehensive approach in diagnosing and managing co-infections involving *Mycobacterium tuberculosis* in regions with low TB prevalence. The absence of a known history of contact with active TB patients raises questions about the possibility of identifying an index TB case in the patient’s environment and highlights the difficulty in determining how the patient developed pulmonary tuberculosis. This suggests the possibility of reactivated latent tuberculosis or recent exposure to an unidentified source, followed by subsequent bacterial superinfection.

In summary, this case sheds light on a rare and challenging clinical scenario involving co-infections with *Mycobacterium tuberculosis*, *Streptococcus pneumoniae*, and *Staphylococcus aureus* in an immunocompetent child. It underscores the need for vigilance in diagnosing and managing such cases, particularly in areas with low TB prevalence where tuberculosis may not be immediately suspected. Clinicians must remain alert to the possibility of pulmonary tuberculosis even in the absence of known contact with a TB patient. An acute presentation of respiratory symptoms with partial radiologic improvement after initial antibiotic therapy should not rule out *Mycobacterium tuberculosis* as a potential pathogen. Although there are no pathognomonic radiologic findings for tuberculosis, apical changes should raise suspicion of pulmonary TB. The complexity of this case highlights the importance of ongoing research and clinical awareness to improve our understanding of such rare presentations and to optimize patient outcomes.
